# Supplementary figures and images for: CCN3 (NOV) Drives Degradative Changes in Aging Articular Cartilage
Source: Int J Mol Sci. 2020 Oct 13;21(20):7556. doi: 10.3390/ijms21207556 (PMC7593953; doi:10.3390/ijms21207556)

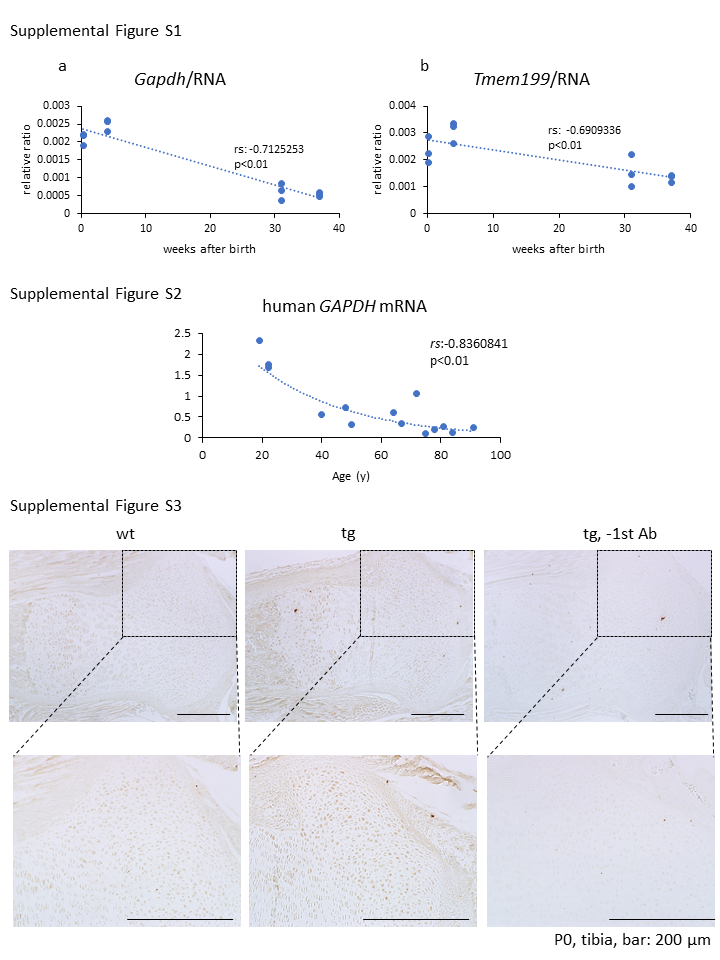

Supplement: Supplementary file 1 [file ijms-21-07556-s001.zip › ijms-938821-supplementary.png]
